# Increased NHE1 expression is targeted by specific inhibitor cariporide to sensitize resistant breast cancer cells to doxorubicin in vitro and in vivo

**DOI:** 10.1186/s12885-019-5397-7

**Published:** 2019-03-08

**Authors:** Qi Chen, Yueqin Liu, Xiao-lan Zhu, Fan Feng, Hui Yang, Wenlin Xu

**Affiliations:** 1 Breast Surgery, the Fourth Affiliated Hospital of Jiangsu University, 20 Zhengdong Road, Zhenjiang, 212001 Jiangsu China; 2 School of Medicine, Jiangsu University, 301 Xuefu Road, Jiangsu, 212013 China; 3 Central Laboratory, the Fourth Affiliated Hospital of Jiangsu University, Zhenjiang, 212001 Jiangsu China

**Keywords:** NHE-1, Cariporide, Sensitivity, MCF-7/ADR, MDR, Chemotherapy

## Abstract

**Background:**

The Na^+^/H^+^ exchanger (NHE1) plays a crucial role in cancer cell proliferation and metastasis. However, the mechanism underlying chemotherapeutic resistance in cancer cells has not been completely elucidated. The NHE1 inhibitor cariporide has been demonstrated to inhibit human cancer cell lines. The goal of this study was to provide new sights into improved cancer cell chemosensitivity mediated by cariporide with activation of the apoptosis pathway.

**Methods:**

The NHE1 expression levels were first evaluated using the online database Oncomine and were determined by RT-PCR and western blot in vitro and in vivo. Cell proliferation was assessed In vitro through a CCK-8 assay, and apoptosis was analyzed by flow cytometry. An in vivo analysis was performed in BALB/c nude mice, which were intraperitoneally injected with MCF-7/ADR cells.

**Results:**

NHE1 levels were significantly higher in breast cancer tissue than adjacent tissue, as well as in resistant cancer cells compared to sensitive cells. Cariporide induced the apoptosis of MCF-7/ADR cells and was associated with the intracellular accumulation of doxorubicin and G0/G1 cell cycle arrest. Moreover, cariporide decreased MDR1 expression and activated cleaved caspase-3 and caspase-9, promoting caspase-independent apoptosis in vitro. In vivo, cariporide significantly improved doxorubicin sensitivity in a xenograft model, enhancing tumor growth attenuation and diminishing tumor volume.

**Conclusions:**

Our results demonstrate that cariporide significantly facilitates the sensitivity of breast cancer to doxorubicin both in vitro and in vivo. This finding suggests that NHE1 may be a novel adjuvant therapeutic candidate for the treatment of resistant breast cancer.

**Electronic supplementary material:**

The online version of this article (10.1186/s12885-019-5397-7) contains supplementary material, which is available to authorized users.

## Background

Breast cancer (BC) is one of the most common female malignant neoplasms [1, 2]. Standard first-line adjuvant chemotherapy of breast cancer contains doxorubicin or paclitaxel, leading to remission of patients in the early stages of BC [3]. Nevertheless, acquired drug-resistance inevitably occurs after several periods of chemotherapeutic treatments [4], possibly due to the expression of ATP binding cassette transporters, resulting in decreasing intracellular drug concentrations, promoting cancer cells proliferation and metastasis [5–7].

NHE1 is an 815-amino acid plasma membrane glycoprotein that is a member of the SLC9A gene family [8]. Increasing evidence had shown that NHE1 plays a crucial role in carcinogenesis, migration, invasion and drug resistance [9]. Previous investigations identified tumor-suppressive effects caused by NHE1 down-regulation in many cancers, such as gastric cancer [10], leukemia [11] and glioma [12], suggesting that it could serve as a therapeutic target for human cancers [13].

There are several NHE1 inhibitors currently available, including amiloride, HMA and cariporide. We focused on cariporide, which has a positive effect on myocardial reperfusion injury [14], and can also attenuate cancer cell proliferation and metastasis [15, 16]. However, the detailed biological functions and underlying molecular mechanisms in drug-resistant breast cancer remain poorly understood. Therefore, the goal of this study was to demonstrate both in vivo and in vitro whether alteration of NHE1 expression can modify breast cancer cell proliferation, apoptosis, and sensitivity to chemotherapeutic drugs. NHE1 inhibitors, such as NHE1shRNA or cariporide, were previously tested in mice and were shown to successfully suppress the development, metastasis and invasion in the transplanted tumors [17, 18]. The inhibitor cariporide used in the study was assayed in vivo and in vitro and was shown to have a similar effect on cancer cell proliferation and apoptosis. We also confirmed that cariporide had a positive effect on altering cancer cell chemo-sensitivity.

## Methods

### Datasets

The gene expression datasets were retrieved from the online Oncomine platform (http://www.oncomine.org/resource/login.html). Statistical analysis of NHE1 expression in The Cancer Genome Atlas [19] dataset, Radvanyi Breast Statistics [20] and in the Zhao Breast Statistics [21] were performed using Student’s t-tests.

### Cell culture

Human breast cancer MCF-7 cells (Stem Cell Bank, Chinese Academy of Sciences, Shanghai, China. Catalogue No: TCHu-74) were cultured in MEM supplemented with 10% fetal bovine serum (FBS), 100 U/ml penicillin and 0.1 mg/ml streptomycin. MCF-7/ADR cells (Shanghai Antique Biotechnology Company, China; Catalogue No: BG006) were grown in RPMI-1640 supplemented as described for MEM with 1 μg/ml doxorubicin (Hisun Pharmaceutical, Zhejiang, 2 mg/ml stock solution in Normal Saline). The human ovarian cancer cell lines A2780 and A2780/PTX were preserved in the Central Laboratory of the Fourth Affiliated Hospital of Jiangsu University, cultured in RPMI-1640 supplemented as described for MEM with 0.8 μg/ml paclitaxel (Oxalcon Pharmaceutical Co., Ltd. Jiangsu, 6 mg/ml stock solution) if when necessary. All cells were incubated under a humidified atmosphere at 37 °C with 5% CO_2_.

### Cancer tissues

Twenty pairs of breast cancer tissues and their matched adjacent breast tissues were obtained from patients who underwent surgery at the Fourth Affiliated Hospital of Jiangsu University. All of the tissues were immediately snap-frozen in liquid nitrogen and stored at − 80 °C until total RNA and protein were extracted. The study protocol was approved, and consent was obtained from all participants in this study.

### Cell proliferation assay

Cells were treated with cariporide (Sigma-Aldrich, Inc., St. Louis, MO, USA, stock in 200 μg/ml in DMSO). Cells were trypsinized and transferred into a 96-well plate at a density of 2 × 10^3^ cells per well, and incubated with drugs at different concentrations of cariporide (3–100 μg/ml), doxorubicin (1–100 μg/ml) and paclitaxel (0.2–30 μg/ml) for 24 h, 48 h. CCK-8 solution (Beyotime Biotechology, Nantong, China, 10 μl in 100 μl of medium) was added to each well, and the cells were incubated for 2 h at 37 °C. The optical density value at 450 nm was determined using a microplate reader (Imark, Lab system; Bio-Rad, USA).

### Fluorescence intensity analysis

Cells were seeded in 6-well plates(1 × 10^4^ cells/well) and attached overnight, and after incubating with 6 and 9 μg/ml cariporide for 48 h, the cells were further treated with doxorubicin(10 μg/ml) at 37 °C for another 2 h. Next, the cells were trypsinized and suspended in 0.5 ml pre-chilled PBS. The mean intensity fluorescence(MIF) were determined by flow cytometry (BD Biosciences, USA) and the data were analyzed with FlowJo version 7.6 (Tree Star Inc., USA).

### Cell cycle and apoptosis analysis

Cells were incubated with cariporide and doxorubicin for 48 h, after which they were stained using an Annexin V/7-AAD staining kit (BD Biosciences, USA). After the Cell cycle analysis, the cells were tested using a Cell Cycle analysis kit (Beyotime, Nantong, China). The apoptosis rate and proportion of cells at different stages of the cell cycle were analyzed by flow cytometry (BD Biosciences, USA). The data was analyzed using FlowJo software version 7.6 (Tree Star Inc., USA).

### Western blot analysis

Total cell lysates containing 100 μg proteins were separated in 8–10% SDS-PAGE gels and transferred to a PVDF membrane. After 2 h of blocking, the membrane was incubated at 4 °C overnight with the primary antibody, either NHE1 (Abcam, USA), MDR1 (Abcam, USA), or caspase-3/9 (Cell Signaling Technology, USA), with β-actin (Cell Signaling Technology, USA) used as a loading control. After washing the membrane with TBST three times, it was incubated with a secondary antibody at a dilution of 1:5000 at room temperature for 1 h. Densitometry was performed using ImageJ(ImageJ 1.48v, National Institutes of Health, USA) to quantify the expression of the protein of interest versus the loading control.

### Reverse transcription and real-time PCR

Total RNA was isolated from cells or tissue using the TRIzol isolation method (Takara Biotechnology Co, Ltd., Dalian, China), with the RNA was subsequently transcribed to cDNA using a PrimeScript RT reagent Kit (Takara, Dalian, China). Real-time polymerase chain reaction (RT-PCR) was performed using a SYBR Green II PCR kit (Takara, Dalian, China) and an ABI7500 (Applied Biosystem Life Technologies, Foster City, CA, USA) thermal cycler. Primers were synthesized by GeneCopoeia Company (FuNeng Gene Company, Guangzhou, China). Differences in gene expression were calculated using the 2^-ΔΔCt^ method.

### Xenograft model in nude mice

Female BALB/c nude mice (5–6 weeks old, body weight 18~22 g) were purchased from the SLAC laboratory animal company (Shanghai, China) and maintained in a specific pathogen-free (SPF) animal lab in Jiangsu University, which was approved by the Laboratory Animal Management Committee of Jiangsu University and met the guidelines of the National Institutes of Health Guide for the Care and Use of Laboratory Animals (Serial No: UJS-LAER-2016101301).

Individual mice were randomized and injected with 5 × 10^6^ MCF-7/ADR (0.2 ml in FBS-free medium) into their left fat pad (*n* = 3 per group). Mice were injected with different drugs every two days 7 times: the NS group received saline (200 μl/animal) as a control; the cariporide (CP) group received cariporide only (3 mg/kg body wt, I.P); the ADR group received doxorubicin (5 mg/kg body wt, I.P); and the combination group received cariporide and doxorubicin together. The growth of implanted tumors was monitored using a Vernier caliper weekly up to 24 days post implantation. The volume of tumors was calculated using the formula: V = ab^2^/2, where “b” is the minimal tumor diameter and “a” refers to the maximum tumor diameter. The day after the last intraperitoneal administration, the mice were sacrificed using the widely accepted method which is cervical spine dislocation and the transplanted tumor tissues were collected, the frozen in liquid nitrogen and stored at − 80 °C for protein and RNA extraction.

### Immunohistochemistry and morphological analysis

For immunohistochemistry (IHC), thin slices were fixed in 4% formaldehyde (pH 7.0) for < 24 h. Tissues were embedded in paraffin, and 3 μm-thick sections were cut and placed onto glass slides coated with 3-aminopropyl-triethoxysilane. The paraffin was removed, the slides were rinsed in distilled water and PBST, and then tissue sections were blocked with 3% peroxide-methanol at room temperature for 20 min. Sections were incubated with primary antibodies for 2 h at 37 °C, and the secondary IgG for 30 min at 37 °C. The compound 3,3-diaminobenzidine (DAB) was added for 10 min at room temperature in the dark, after which hematoxylin dye was added. Sections were then dehydrated with neutral glue, cleaned and fixed. Protein expression was classified semi-quantitatively using Image Pro Plus (version 6.0).

### Statistical analysis

The experimental data are presented as the means ± standard deviation. All statistical analyses were performed using GraphPad Prism 5(GraphPad Software, La Jolla, CA, USA) and determined by unpaired two-tailed Student’s t-test and two-way analysis of variance analyses. Differences were considered significant at *P* < 0.05.

## Results

### NHE1 expression varies in breast cancer tissue and cell lines

**Fig. 1:**
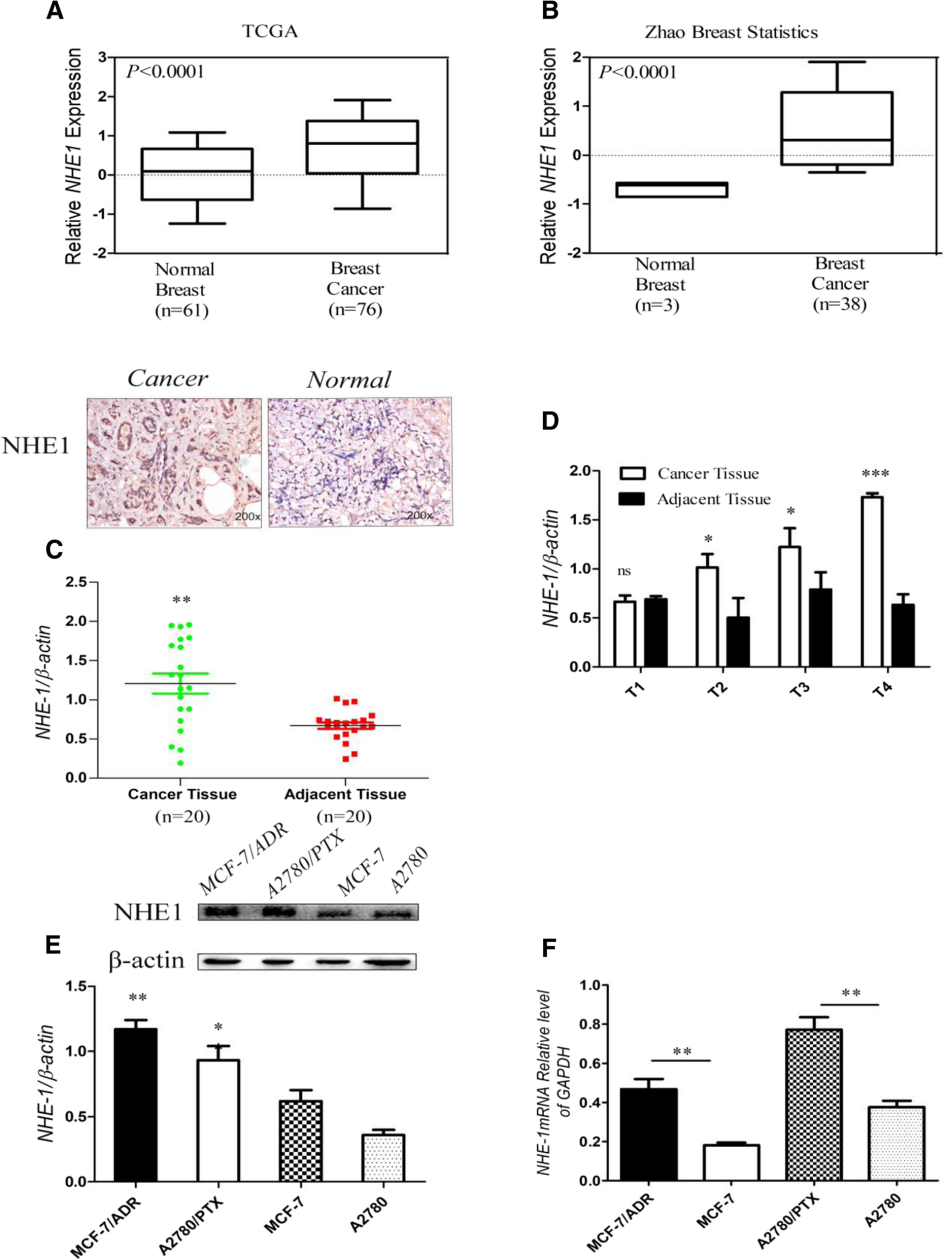
Expression of NHE1 is altered in human breast cancer. (**a**) NHE1 expression in normal versus breast cancer tissues using publicly available datasets from The Cancer Genome Atlas (TCGA). (**b)** Zhao breast statistics. (c) Immunohistochemistry and western blot analysis of NHE1 protein expression in 20 paired primary breast cancer tissues and their corresponding adjacent tissues. (**d**) Western blot analysis of NHE-1 in different pathological stages. (e, f) Expression of NHE1 in sensitive MCF-7 and A2780 cell lines and drug-resistant MCF-7/ADR and A2780/PTX cells was assessed by RT-PCR and western blot. All graphs show the means±SD of three independent experiments

To verify the crucial role of NHE1 in breast cancer, we examined publicly available gene expression datasets, and observed that NHE1 is up-regulated in the majority of breast tumors in three independent datasets (see Additional file 1: Figure S1A, and Fig. 1a, b), and was associated with poor prognosis (Fig. S1B, S1C). Immunohistochemistry analyses were performed on 20 female breast carcinoma samples and their adjacent breast tissues. As shown in Fig. 1c and summarized in Fig. 1d, NHE1 was absent in para-cancerous tissues. Strong positive staining gradually increased from ductal carcinoma in situ to invasive ductal carcinoma, which is in accordance with previous findings showing that NHE1 mRNA expression is elevated in invasive breast cancer [22]. The expression of NHE1 was associated with different types and stages of cancer cells [23]. In addition, we detected NHE1 expression in sensitive cells and chemoresistant cell lines, including breast cancer(MCF-7 and MCF-7/ADR) and ovarian cancer(A2780 and A2780/PTX) by western blot analysis(Fig. 1e) and RT-PCR(Fig. 1f). The results reveal that NHE1 expression is much higher in drug-resistant cells than that in sensitive cells, which is due to the different origins and resistance statuses of tumor cells. Taken together, these observations suggest that NHE1 may promote the chemo-resistance of breast cancer.

### Cell proliferation and viability are altered by cariporide

**Fig. 2:**
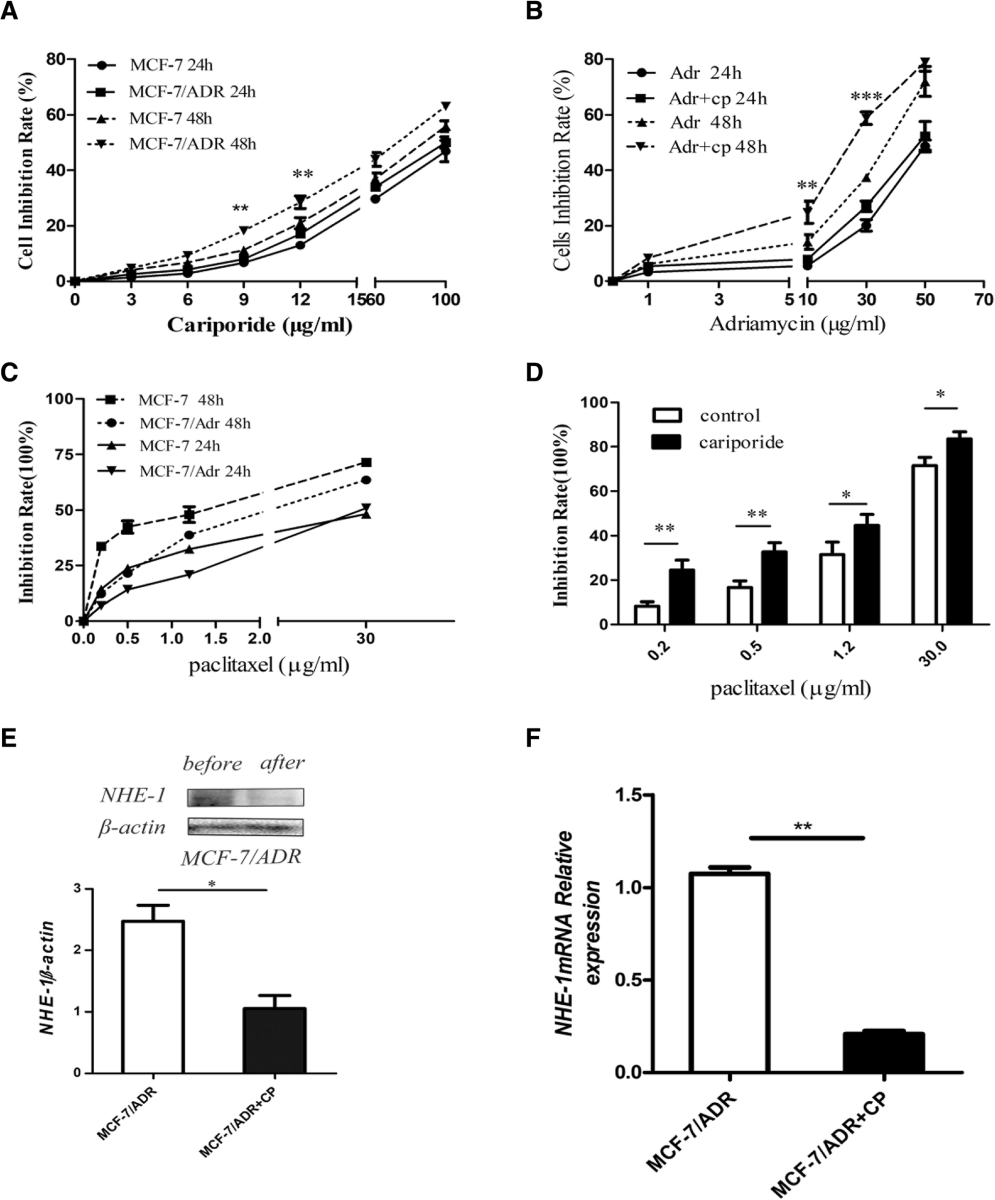
Cariporide sensitizes resistant breast cancer cells to chemotherapeutic drugs. (**a**) Cells were treated with a range of cariporide concentrations(3-100 μg/ml) for 24 and 48 h, and the OD value was measured for the CCK8 assay. We chose 6 μg/ml cariporide as the combined-treatment concentration in the follow-up experiments. (**b**) Cells were treated in the presence or absence of 6 μg/ml cariporide and doxorubicin (1, 5, 50, 100 μg/ml) at the indicated time points and were tested using a CCK8 assay. (**c**) Cells were treated with different concentrations (0.2, 0.5, 1.2, 30 μg/ml) of paclitaxel for 24 and 48 h and were tested using a CCK8 assay. (**d**) MCF-7//ADR cells were treated with the combination treatment of cariporide (6 μg/ml) and paclitaxel (0.2, 0.5, 1.2, 30 μg/ml) for 48 h, and cell proliferation was detected using a CCK-8 assay. (**e**) MCF-7/ADR cells were incubated with 6 μg/ml cariporide for 48 h, NHE1 protein expression was detected by western blot, and (**f**) NHE1mRNA was confirmed by RT-PCR at 24 h. Cycling conditions were as follows: 95 °C for 10 min, followed by 40 cycles at 95 °C for 15 s and 60 °C for 45 s, with a final extension step of 95 °C for 15 s, 60 °C for 20 min, 95 °C for 15 s, and 60 °C for 15 s

Cariporide significantly decreased NHE1 expression in breast cancer cells (Fig. 2e, f), where it inhibited the proliferation of MCF-7 and MCF-7/ADR cells in a dose- and time- dependent manner, as assessed by CCK8 assays with a range of concentrations (Fig. 2a), of doxorubicin (Fig. 2b) or paclitaxel (Fig. 2c) in culture for 24 and 48 h. In addition, after cotreatment with cariporide and doxorubicin, the IC50 value decreased to 17.16 ± 0.06 μg/ml(2.463-fold), which was significantly lower than in cells treated with doxorubicin only. The same results were observed in the paclitaxel-only and cotreatment groups (Fig. 2c, d). These results suggest that cariporide can sensitize drug-resistant cells to chemotherapeutic drugs after the cotreatment with cariporide and doxorubicin or paclitaxel.

### Cariporide induces cell apoptosis and modulates the cell cycle

**Fig. 3:**
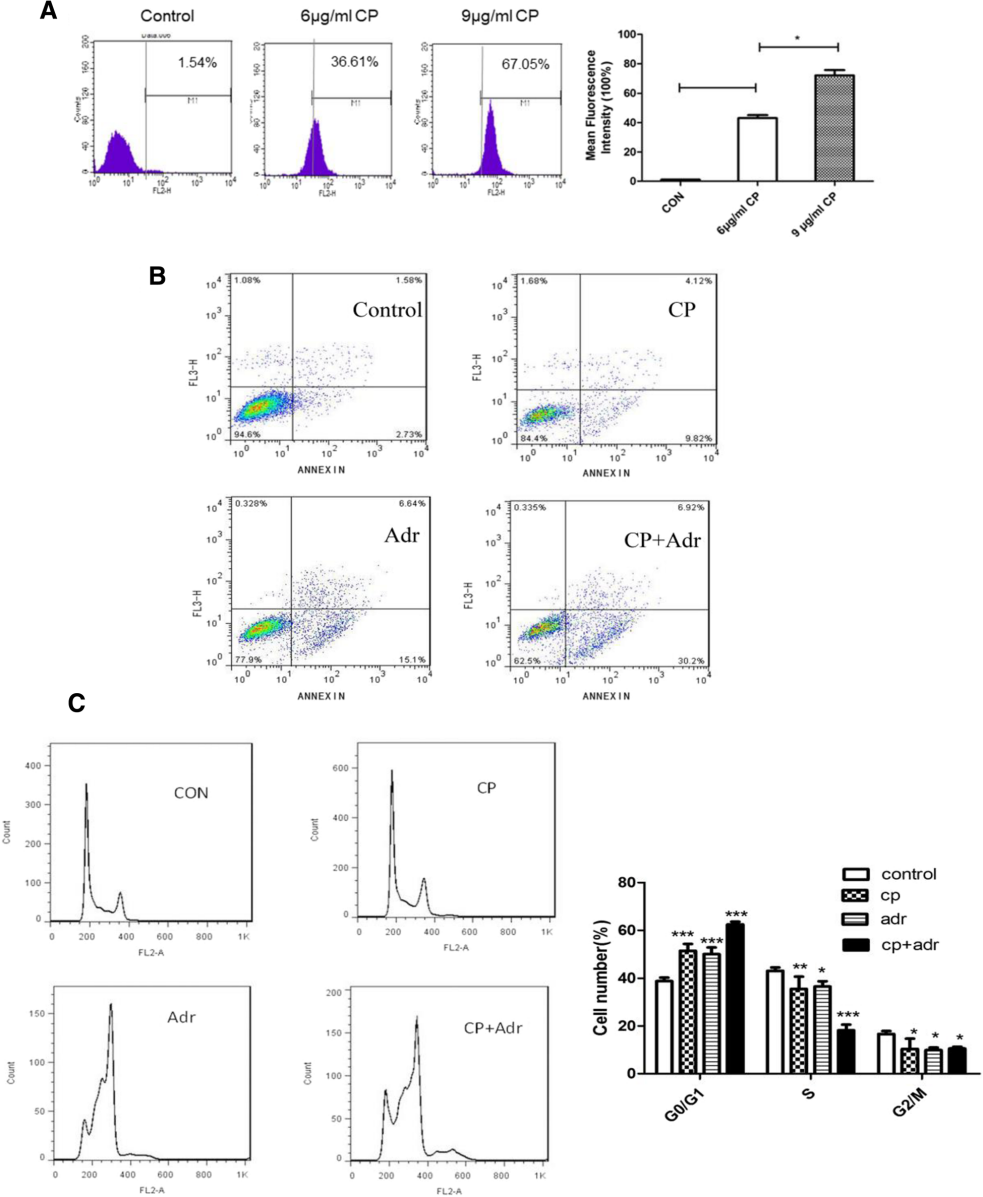
Cariporide enhances doxorubicin-induced apoptosis in MCF-7/ADR cells. (**a**) Cells were either untreated (control) or pretreated with 6 or 9 μg/ml cariporide (CP) alone for 48 h, then each group was incubated with doxorubicin (10 μg/ml) for 2 h. Subsequently, and the fluorescence intensity of doxorubicin was detected by flow cytometry. Bar graph and error bars represent the means and standard deviation (SD), respectively. *P < 0.05. The analysis was performed in triplicate in two independent experiments. (**b**) Cells were harvested after treatment with 6 μg/ml cariporide (CP) and 10 μg/ml doxorubicin (Adr) alone or together for 48 h and then were stained using the AnnexinV/7-AAD kit and analyzed by flow cytometry. Early apoptotic cells lie in the lower right quadrant. (**c**) Cells were either untreated or treated with 6 μg/ml cariporide (CP) alone or with doxorubicin (Adr, 10 μg/ml) for 48 h, then they were harvested and fixed in 70% ethanol at 4 °C overnight. Subsequently, the percentage of each phase of the cell cycle was determined by flow cytometry

Doxorubicin is a chemotherapy drug that can damage the structure of DNA, altering of biological processes, such as apoptosis and necrosis [24]. We sought to determine whether the rate of cariporide-induced ADR-uptake would result in an enhanced sensitivity of MCF-7/ADR cells to doxorubicin treatment. The mean fluorescence intensity was enhanced with cariporide pretreatment (Fig. 3a), and as the concentration of cariporide increased, so did the fluorescence intensity. In addition, we surprisingly detected apoptosis induction in the combination group (Fig. 3b), indicating that cariporide can enhance doxorubicin-induced apoptosis. As shown in Fig. 3c, cariporide and doxorubicin had a significant positive effect on doxorubicin-induced accumulation of cells in the G0/G1 phase of the cell cycle, although it appeared that cariporide alone lowered the percentage of cells in S phase. Furthermore, the diploid apoptotic peak was clearly higher in the cotreatment group before the G0/G1 peak. Taken together, these findings suggest that cariporide promotes cell apoptosis and modulates the cell cycle in vitro.

### Cariporide activates the apoptosis pathway

**Fig. 4:**
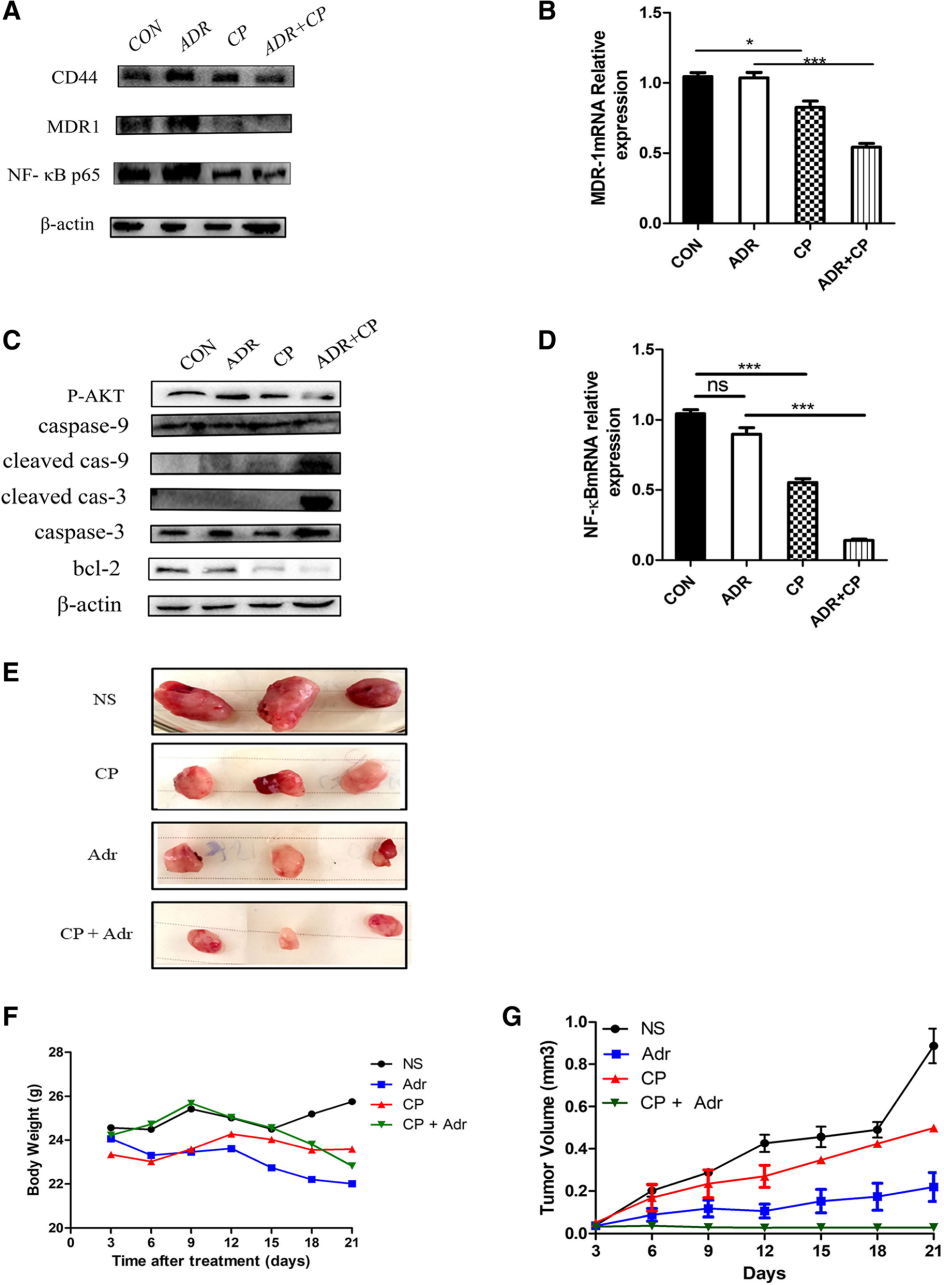
Cariporide activates the doxorubicin-induced apoptosis pathway. (**a**) Cells were either untreated or pretreated with 6 μg/ml cariporide (CP) and 10 μg/ml doxorubicin (ADR) alone for 48 h. The combination group contained cariporide (6 μg/ml) for 24 h followed by addition of doxorubicin (10 μg/ml) for another 24 h. The whole cell lysates were immunoblotted for MDR1, CD44, NF-κB p65 and (**c**) p-AKT, cleaved caspase 3/9, and Bcl-2. (**b**) MCF-7/ADR cells were treated with 6 μg/ml cariporide and/or 10 μg/ml doxorubicin for 24 h. The cell isolations were prepared for MDR1 mRNA and (**d**) NF-κB mRNA subjected to RT-PCR analysis. (**e**) Female nude mice were randomized and inoculated with MCF-7/ADR cells (5 × 10^6^/ml), and the growth of implanted breast tumors was monitored every week after inoculation until the volume increased to 1 cm^3^. Xenograft tumors were treated with either cariporide (3 mg/kg) or doxorubicin (5 mg/kg), or their combination every two days for a total of seven times. On day 21 posttreatment, all animals (*n* = 20) were sacrificed, and the tumors were removed and measured. The data represent the means ± SD from at least 3 animals per group. (**f**) Body weight and tumor size were measured twice a day using calipers, and (**g**) tumor volumes were plotted

Apoptosis is a form of programmed cell death that is regulated by Bcl-2 and specific apoptotic mediators(Caspase-3/9, Bcl-2 and PARP) [25]. We observed that MCF-7/ADR cells that were pretreated with cariporide and doxorubicin exhibited markedly increased levels of Caspase-3/9 cleavage, which was accompanied by Bcl-2 reduction(Fig. 4c). The p-AKT expression was decreased after the CP induction, which was extremely down-regulated in the combination group(Fig. 4c). We concluded that NHE1 down-regulation reduced cell proliferation due to the activation of the apoptosis signaling pathway [26],and it may possibly suppressed the PI3K/AKT signaling pathway. Interestingly, CD44 expression was also decreased after cells were treated with cariporide alone, which did not occur in the ADR-treated group. The same results were observed for MDR1 and NF-κB p65 expression, as shown in Fig. 4a. Moreover, under the same conditions, MDR1 and NF-κB p65 mRNA levels also decreased(Fig. 4b, d). Thus, cariporide has the potential to be a highly promising and truly effective activator of the apoptosis pathway, acting as an anticancer agent in breast cancer cells.

### Cariporide attenuates tumor growth and induces apoptosis in nude mice

Evidence has shown that the down-regulation of NHE1 is a trigger for activation of the apoptosis pathway due to proliferation inhibition in different types of cancer [27]. We next evaluated whether cariporide exhibited the same effects in vivo. After continuous intraperitoneal injection of cariporide (3 mg/kg) for 7 days, the body weight and behavior of nude mice showed no significant toxic effects. However, cariporide significantly retarded the growth of tumors in vivo (Fig. 4e). The tumor volumes and weights significantly decreased in the two cariporide-treated groups compared to the other assayed groups (Fig. 4f, g). In detail, no significant difference in the body weight of each group was detected initially, but decreases in body weight were observed 15 days after administration, and even the control group and single ADR group were significantly lower on day 21 (Fig. 4f). These data suggest that NHE1 is an upstream effector of the process of cariporide-induced inhibition of breast cancer cell proliferation.

**Fig. 5:**
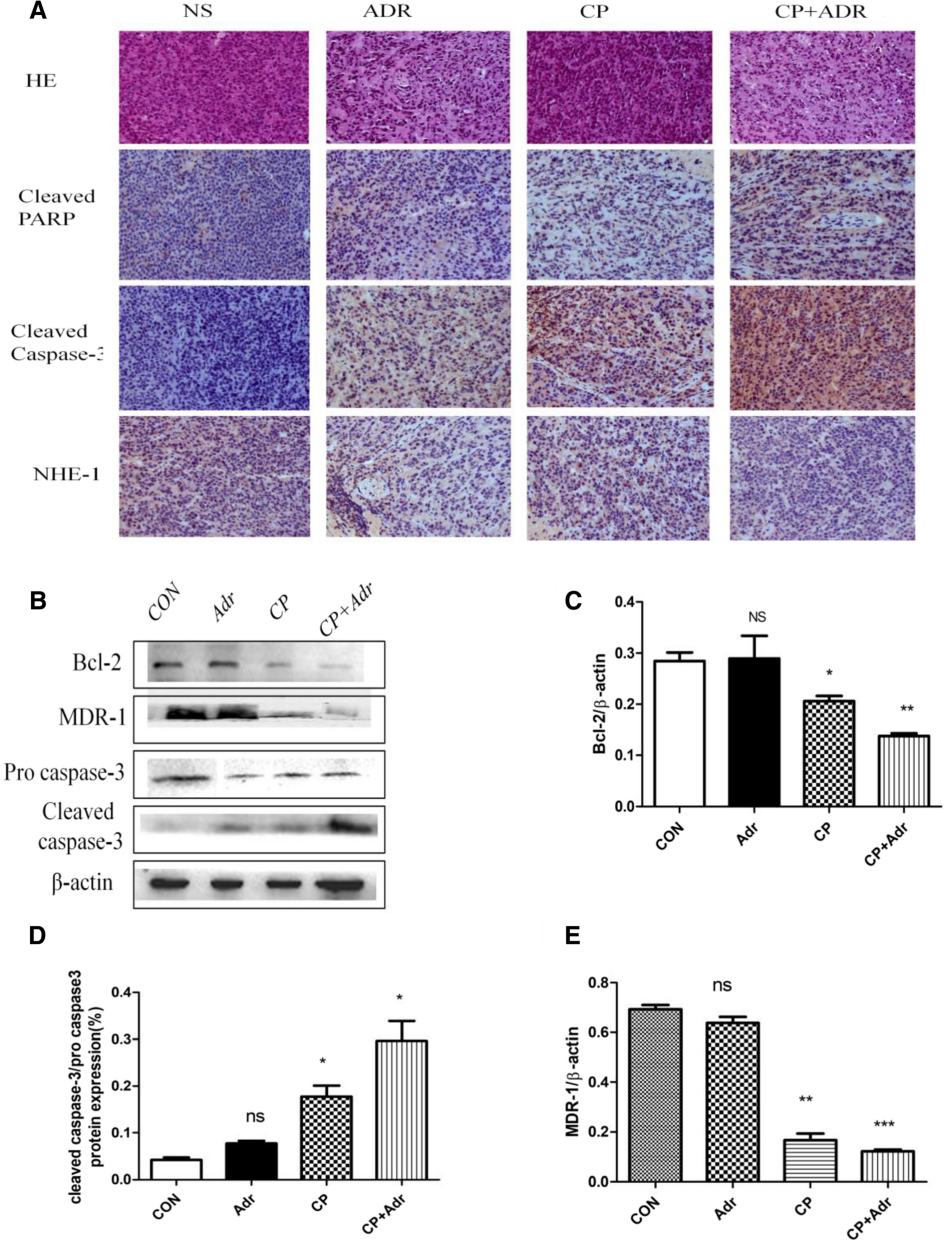
Cariporide enhances the sensitivity of cancerous tumors to doxorubicin in vivo. (**a**) Tumor tissues from each group were stained with H&E, while NHE1, cleaved PARP and caspase-3 expression in the tumor sections was characterized by immunohistochemistry. (**b**) Tissue lysates were subsequently prepared and subjected to western blotting analysis using the indicated antibodies. (**c**-**e**) Relative expression of MDR1, Bcl-2, and cleaved caspase-3 is shown in the histogram

Accompanied by the observed decrease in NHE1, the immunohistochemistry analysis revealed that cleaved caspase-3 and PARP expression in the combination group were significantly higher than in the control group (Fig. 5a). In addition, histological analysis of the combination group clearly showed loose arrangements of cells, and a wide range of degeneration and necrotic areas (Fig. 5a). CD44 is closely related to tumor resistance, and the CD44^+^/CD24^−^ phenotype may be an important factor of malignant relapse with chemoresistance in patients with surgically resected invasive ductal carcinoma [28]. However, no obvious differences of CD44 levels in the necrotic areas in the single-treated groups were observed. Moreover, the combination group also showed a significant decrease in MDR1 and Bcl-2 expression, as well as higher levels of cleaved PARP and cleaved caspase-3 (Fig. 5b). Nevertheless, the doxorubicin-treated group failed to induce apoptosis, which is primarily attributed to the lower doses of doxorubicin (5 mg/kg) being unable to induce the apoptosis threshold of the resistant cells. Collectively, cariporide inhibited the growth of implanted breast cancer and increased its sensitivity to doxorubicin in nude mice.

## Discussion

Although chemotherapy plays a major role in the treatment of breast cancer, acquired drug resistance limits its effectiveness and affects patient’s prognosis. In studies performed to identify ways to manipulate cancer cell resistance, NHE1 has been shown to be differentially expressed in several types of cancers and may potentially regulate drug resistance. In this study, cariporide, a non-amiloride derivative, was used to inhibit the proliferation of MCF-7 and MCF-7/ADR cells and ultimately induced cell apoptosis. Indeed, down-regulation of NHE-1 expression or inhibition of its activity, inhibited intracellular hyperacidity, tumor cell growth stagnation and selective apoptosis [29]. However, the mechanism of action leading to apoptosis induced by cariporide is far from elucidated.

Previous studies showed that NHE1 mediates the migration and invasion of tumor cells [30], as cariporide had been shown to reduce the expression of NHE1-mediated proliferation of human carcinoma cells, such as tongue cancer cells [31] and MDA-MB-231 cells [32]. Most studies of amiloride derivatives or NHE1siRNA- transfected cells have been demonstrated to lead to NHE1 reduction in specific types of cancers, but the function of cariporide in breast cancer has not been widely investigated. Currently, several studies have focused on cariporide, which originates from non-amiloride-derivatives, showing that it suppressed SASL1m metastasis in vivo and reduced the invasive activity of SASL1m cells [17]. Importantly, cariporide has been clinically tested in cardiology and ischemia-reperfusion injury, which demonstrated it to be useful in cardiovascular disease treatments [33–35].

In this study, we aimed to determine the functional effect leading to breast cancer cell apoptosis, which occurred after the combined treatment of cariporide and doxorubicin. A previous study demonstrated that the combination index (CI) of cariporide and doxorubicin was less than one [36], indicating that they have synergistic effects. In this study, we focused on studying the alteration of the IC50 value, which was significantly decreased after the combined treatments.

Based on abundant evidence, NHE1 expression is altered in tumors compared to in normal tissues [15, 37–39], and NHE1 expression can be stimulated by serum-free media to regulate the intracellular microenvironment during chemotherapy in cancer cells, which cannot be achieved in normal cells. Moreover, NHE1 upregulation due to miR-27b, leads to the proliferation of cervical cancer cells [40]. Furthermore, enhanced sensitivity of the resistant cells to Imatinib occurs by activating the p38/MAPK pathway [41], which may directly verify that NHE1 is correlated to tumorigenesis, pathological remodeling, invasion and metastasis, and chemotherapy and immunotherapy drug resistance [42]. In our study, we concluded from analysis of the public datasets, such as TCGA, that NHE1 expression is higher in cancer than in paracancerous tissues, and that higher expression of NHE1 indicated poor survival. We also confirmed that higher NHE1 expression occurs in MCF-7/ADR cells, indicating a close relationship between NHE1 and drug resistance. Apparently, the expression of NHE1 forces cancer cells to participate in tumor-specific apoptosis [43].

Caspase-9 is activated by mitochondrial dysfunction, which is followed by cleavage caspase-9 expression [44], which is an initiator protein of intrinsic apoptotic pathways, involving the abnormal expression of Bcl-2 family proteins, such as Bax and Bcl-2 [45]. Aberrant activation of Bcl-2 and caspase3/9 expression was associated with apoptotic pathway activation [46–48]. This result was consistent with observations in K562 cells and in MCF-7 cells with NHE1 inhibition by amiloride derivatives [49], and we verified the induction of apoptosis in breast cancer cells treated with cariporide, and even higher apoptosis rates were observed after the addition of doxorubicin. Furthermore, our data showed the expression of Bcl-2 decreased in response to the cariporide treatment, and was further down-regulated after the combined-treatment in vitro and in vivo, whereas cleaved caspase-3/9 expression was activated to drive the apoptosis process.

Doxorubicin primarily affects the chemical structure of DNA, leading to growth retardation in the G0/G1 phase of tumor cells [50]. In this study, we confirmed that cariporide and doxorubicin together further increased the proportion of MCF-7/ADR cells in the G0/G1 phase, while the number of cells in the S phase partially decreased. Furthermore, the efflux of doxorubicin was severely decreased. These results show exceptional synergistic effects of the drug combination on the cell cycle and apoptosis.

P-glycoprotein (P-gp), is an ATP-dependent membrane pump, that is positively correlated with drug resistance [51, 52]. A worldwide effort has been made to study the function of P-gp transporters and reduce multiple drug resistance (MDR), which promotes the accumulation and sensitivity of chemotherapeutic drugs [53]. Surprisingly, we observed that cariporide could decrease the expression of MDR-1, CD44, and NF-κB in vitro. However, no significant difference was observed between the doxorubicin-alone and the control groups, primarily because MCF-7/ADR cells are inherently resistant to doxorubicin. NF-κB was shown to improve the anti-apoptotic activity of tumor cells by regulating the transcription of Bcl-2, leading to drug resistance of tumor cells [54]. Our study further showed that the cotreatment surprisingly increased caspase-independent apoptosis as well as leading to decreased expression of p-AKT and the resistant proteins in vitro and in vivo. Furthermore, cariporide and doxorubicin activated cleaved PARP and cleaved caspase-3 expression in transplanted tumor tissues. Thus, the two drugs clearly have synergistic effects, providing further support for the notion that NHE1 may be a valuable target for treating breast cancer cells, possibly via the PI3K/AKT signaling pathway.

In this study, we showed that high NHE1 expression was associated with chemoresistance. Cariporide inhibited breast cancer cell proliferation and activated apoptosis, supporting the notion that targeting NHE1 is likely a valuable therapeutic strategy for resistant breast cancer. Taken together, these data confirmed that cariporide may serve as a chemotherapy sensitizer. Of note, our findings may provide new insights into NHE1 association with MDR and activation of NF-κB p65 signaling and the caspase signal pathway.

## Conclusions

In summary, our results demonstrated that the expression of NHE1, is negatively regulated by cariporide, and can likely serve as an independent hallmark of cancer prognosis in patients, since NHE1 is closely related to the chemoresistance of breast cancer. Targeting NHE1 attenuated cell proliferation, as well as induced apoptosis, which were accompanied by MDR1 reduction and caspase-3 pathway activation, respectively. Collectively, we suggest that NHE1 may serve as a promising target, and an understanding of the molecular mechanism activated by cariporide could represent a promising adjuvant intervention to improve the outcome of chemoresistant breast cancer therapy.

## Additional files

Additional file 1: **Figure S1.** NHE1 is upregulated in human breast cancer databases and is correlated to cancer prognosis. (A) Meta-analysis of NHE1 expression in three different publicly available datasets from The Cancer Genome Atlas (TCGA), Radvanyi Breast Statistics and Zhao breast statistics. (B) Correlation between NHE1 levels and patient disease prognosis in the ER(+) patient cohort. The vertical axis is the patient survival rate, while the observation days are shown on the abscissa. (C) Correlation between NHE1 level and patient’s disease prognosis in ER(−) patients cohort. The vertical axis is the patient’s survival rate, while abscissa for the observation days. (PDF 317 kb)

## Abbreviations

ErbB2: Erb-B2 Receptor Tyrosine Kinase 2
MDR1: Multiple Drug Resistance-1
NHE1: Na^+^/H^+^ Exchanger 1
PARP: poly ADP-ribose polymerase
SLC9A family: Solute carrier family 9 member A
TCGA: The Cancer Genome Atlas
