# Supplementary material for: Increased NHE1 expression is targeted by specific inhibitor cariporide to sensitize resistant breast cancer cells to doxorubicin in vitro and in vivo
Source: BMC Cancer. 2019 Mar 8;19:211. doi: 10.1186/s12885-019-5397-7 (PMC6408845; doi:10.1186/s12885-019-5397-7)
Supplement: Supplementary file 1 — Figure S1. NHE1 is upregulated in human breast cancer databases and is correlated to cancer prognosis. (A) Meta-analysis of NHE1 expression in three different publicly available datasets from The Cancer Genome Atlas (TCGA), Radvanyi Breast Statistics and Zhao breast statistics. (B) Correlation between NHE1 levels and patient disease prognosis in the ER(+) patient cohort. The vertical axis is the patient survival rate, while the observation days are shown on the abscissa. (C) Correlation between NHE1 level and patient’s disease prognosis in ER(−) patients cohort. The vertical axis is the patient’s survival rate, while abscissa for the observation days. (PDF 317 kb) [file 12885_2019_5397_MOESM1_ESM.pdf]

A

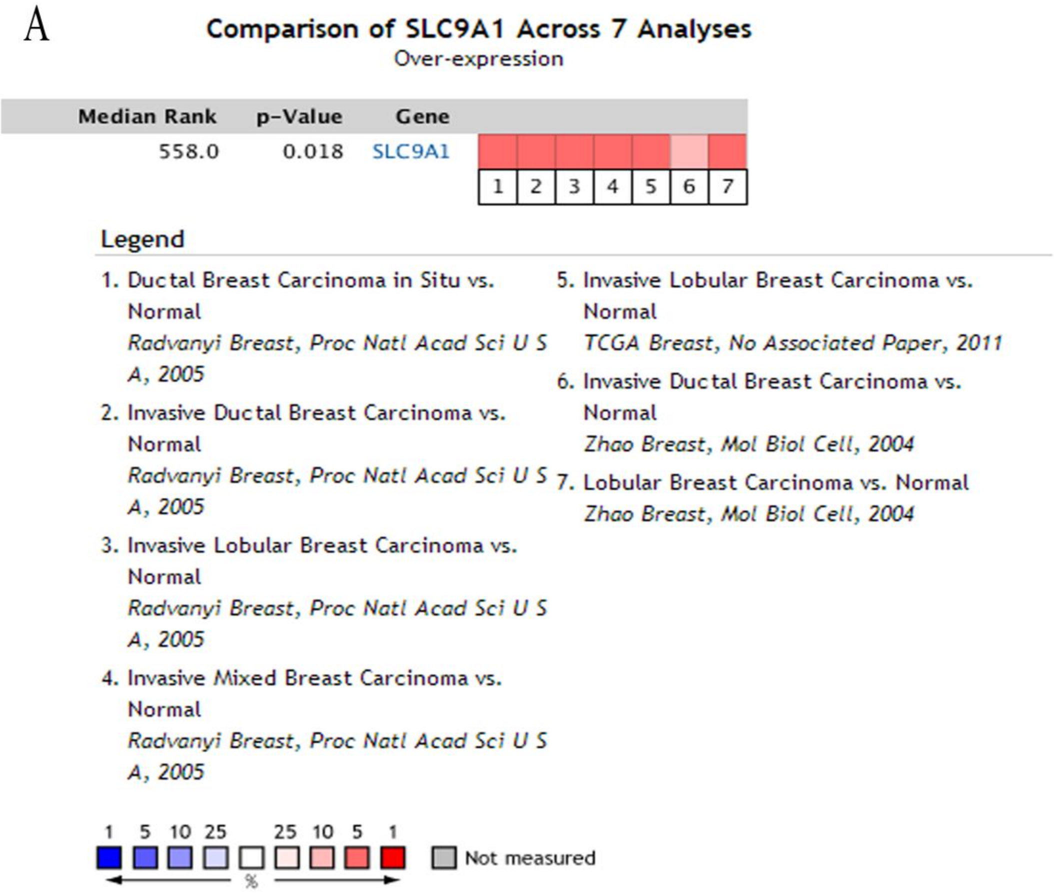
Comparison of SLC9A1 Across 7 Analyses
Over-expression
Median Rank
p-Value
Gene
558.0
0.018
SLC9A1
1 2 3 4 5 6 7
Legend
1. Ductal Breast Carcinoma in Situ vs. Normal
Radvanyi Breast, Proc Natl Acad Sci U S A, 2005
2. Invasive Ductal Breast Carcinoma vs. Normal
Radvanyi Breast, Proc Natl Acad Sci U S A, 2005
3. Invasive Lobular Breast Carcinoma vs. Normal
Radvanyi Breast, Proc Natl Acad Sci U S A, 2005
4. Invasive Mixed Breast Carcinoma vs. Normal
Radvanyi Breast, Proc Natl Acad Sci U S A, 2005
5. Invasive Lobular Breast Carcinoma vs. Normal
TCGA Breast, No Associated Paper, 2011
6. Invasive Ductal Breast Carcinoma vs. Normal
Zhao Breast, Mol Biol Cell, 2004
7. Lobular Breast Carcinoma vs. Normal
Zhao Breast, Mol Biol Cell, 2004
1 5 10 25 25 10 5 1
%
Not measured

B

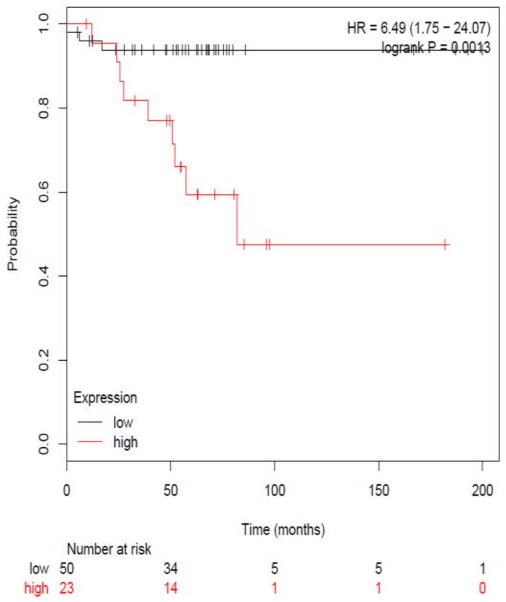
HR = 6.49 (1.75 − 24.07)
logrank P = 0.0013
Probability
0.0
0.2
0.4
0.6
0.8
1.0
Expression
low
high
0
50
100
150
200
Time (months)
Number at risk
low 50 34 5 5 1
high 23 14 1 1 0

C

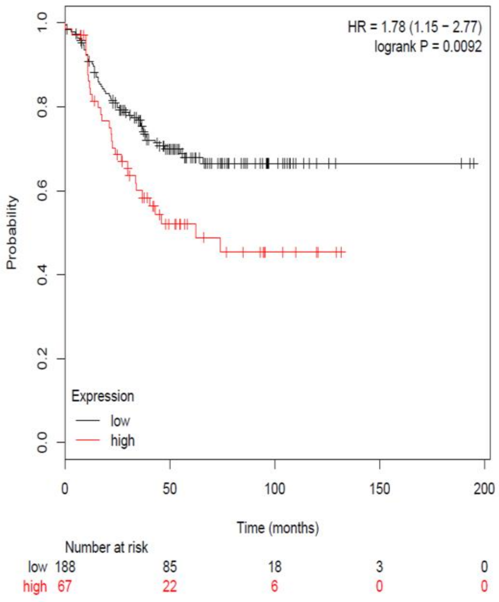
HR = 1.78 (1.15 − 2.77)
logrank P = 0.0092
Probability
0.0
0.2
0.4
0.6
0.8
1.0
Expression
low
high
0
50
100
150
200
Time (months)
Number at risk
low 188 85 18 3 0
high 67 22 6 0 0
